# Self-collected versus clinician-collected human papillomavirus samples

**DOI:** 10.1590/1806-9282.20260122

**Published:** 2026-07-20

**Authors:** Ramazan Bülbü, Jule Eriç Horasanlı, Arif Caner Erdoğan, Fatih Akkuş

**Affiliations:** 1 Aksaray Training and Research Hospital – Aksaray, Turkey.; 2 Necmettin Erbakan University, Faculty of Medicine – Konya, Turkey.; 3 Ali Kemal Belviranlı Obstetrics and Gynecology and Children's Hospital – Konya, Turkey.; 4 Kütahya City Hospital, Department of Obstetrics and Gynecology – Kütahya, Turkey.

**Keywords:** Uterine cervical neoplasms, Mass screening, Human papillomavirus viruses, Vaginal smears

## Abstract

**OBJECTIVE:**

The aim of this study was to evaluate the comparability of self-collected and clinician-collected samples for human papillomavirus detection, cytological findings, and identification of infectious agents.

**METHODS:**

This prospective controlled clinical trial enrolled 124 women aged 30–65 years due for routine cervical cancer screening. This prospective comparative study enrolled 124 women aged 30–65 years who were eligible for routine cervical cancer screening. Participants were allocated to either a clinician-collected control group (n=51) or a self-collected group (n=73) via systematic allocation based on clinic registration order. Viral deoxyribonucleic acid analysis was performed by multiplex real-time polymerase chain reaction; cytological evaluation followed the Bethesda classification system. Infectious agents and participant satisfaction were also assessed. Laboratory personnel were blinded to group allocation.

**RESULTS:**

Overall human papillomavirus positivity was 21.8% (27/124), with rates of 27.5% (14/51) in the control group and 17.8% (13/73) in the self-collected group (p=0.200). Cytological abnormalities were detected in 5.5% of self-collected samples (three cases of atypical squamous cells of undetermined significance, one case of atypical squamous cells cannot exclude high-grade squamous intraepithelial lesion) versus 0% in the control group (p=0.236). Inflammatory smear findings, *Candida* (9.8 vs. 8.2%), and *Trichomonas vaginalis* (0 vs. 1.4%) detection rates were similar between groups. Mean satisfaction score was 12.6±1.62 out of 16 (78.8% positive responses).

**CONCLUSION:**

Self-collected human papillomavirus sampling yields results diagnostically comparable to clinician-collected specimens. Self collected human papillomavirus sampling demonstrated similar human papillomavirus detection rates compared with clinician-collected specimens. Given its feasibility and high acceptability, self-collection may serve as an effective complementary approach in cervical cancer screening programs, particularly for reaching women who have not previously participated in screening.

## INTRODUCTION

Cervical cancer remains a significant global health burden and ranks as the fourth most common cancer among women worldwide^1^. Virtually all cervical cancers arise from infection with carcinogenic types of human papillomavirus (HPV)^2^. Primary prevention through HPV vaccination has been successfully implemented in many high-income countries; however, vaccine coverage remains inadequate in low- and middle-income settings. HPV deoxyribonucleic acid (DNA) screening is therefore essential for reducing cervical cancer morbidity and mortality in regions where vaccination has not been widely adopted^3^.

HPV testing has demonstrated superior reliability and sensitivity compared with conventional cytology, with established reproducibility that permits extended screening intervals^4^. Many countries have adopted HPV-based screening programs^5^. In Turkey, HPV screening begins at age 30 and continues at five-year intervals until age 65^6^.

Self-sampling for HPV detection offers several advantages over clinician-collected specimens. In addition to its practical benefits, vaginal self-sampling has been well accepted across age ranges, albeit younger women may need more targeted reassurance and education to overcome pain and doubt^7^. This approach is less costly, eliminates the need for gynecological examination, and facilitates sample collection in resource-limited settings and hard-to-reach populations^8^. Nevertheless, consensus regarding the optimal sampling method for diagnostic performance remains undefined. Clinician-collected samples obtained during speculum examination remain the reference standard^9^.

This study compared self-collected and clinician-collected HPV samples. The objective was to evaluate the effectiveness and acceptability of HPV screening methods and to provide data that may inform improvements to cervical cancer screening programs in Turkey.

## METHODS

### Study design and ethics

This prospective controlled clinical trial received approval from the institutional ethics committee (Ethics Committee Decision No: 2023/4712, Date: December 15, 2023). This prospective comparative study received approval from the institutional ethics committee (Ethics Committee Decision No: 2023/4712, Date: December 15, 2023). The study conforms to recognized standards, including the Declaration of Helsinki. Written informed consent was obtained from all participants prior to inclusion.

### Participants

**Figure 1:**
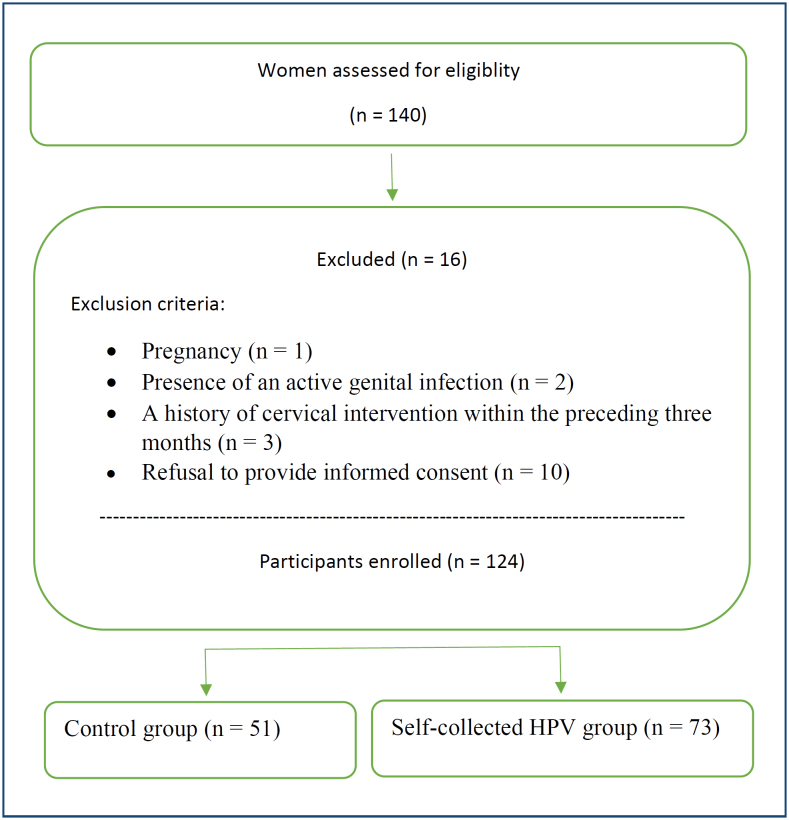
Flow diagram illustrating participant selection, exclusions, and allocation to study groups. HPV: human papillomavirus.

Women who presented to the gynecology outpatient clinic of Necmettin Erbakan University Hospital for any reason between January 2024 and January 2025 and were due for routine cervical cancer screening were enrolled. Eligible participants were women aged 30–65 years who provided written informed consent and had no prior documented HPV positivity. Exclusion criteria were ongoing pregnancy, presence of an active genital infection, a history of cervical intervention within the preceding three months, and refusal to provide informed consent. The participant selection process, exclusions, and allocation to the study groups are illustrated in a Consolidated Standards of Reporting Trials-style flow diagram (Figure 1).

### Group allocation and blinding

Participants were assigned to one of two study groups via a systematic allocation based on clinic registration order: odd numbers were assigned to the clinician-collected sampling group (control) and even numbers to the self-collected group. This method was deterministic and did not allow for allocation concealment, therefore it does not meet the criteria for true randomization. The self-collected group experienced higher enrollment because participants perceived this non-invasive method as more acceptable, which resulted in unequal group sizes, consistent with the pragmatic nature of the study design. Because the allocation was based on clinic registration order rather than true randomization, the study design should be interpreted as a prospective comparative study rather than a randomized clinical trial. Laboratory personnel analyzing samples were blinded to group allocation.

### Sample collection procedures

Clinician-collected sampling (control group): With participants in the lithotomy position, a cervical sample was obtained under direct visualization via a cervical brush following speculum insertion. No antiseptic solution was applied. The sample was placed in a transport medium and sent to the laboratory.

Self-collected sampling (self-HPV group): Prior to sample collection, participants received standardized instruction comprising video demonstration and verbal explanation with an anatomical model. The video detailed the sampling technique, proper brush handling, and key procedural considerations. Participants then collected cervicovaginal samples utilizing an HPV brush in their preferred location—examination room, restroom, or home. Samples were placed in a transport medium and transported to the laboratory.

### Laboratory analysis

Cervical samples were processed in the pathology laboratory through standard cytological staining methods. Findings were reported according to the Bethesda classification system. HPV DNA analysis was performed via the Genematrix NeoPlex™ HPV29 Kit with multiplex real-time polymerase chain reaction. HPV genotypes were classified as high-risk or low-risk types.

### Participant satisfaction assessment

A structured questionnaire developed by the investigators was administered to assess post-procedure satisfaction. It comprised eight items designed to evaluate participant experiences with self-sampling, covering comfort during the procedure, confidence in sample quality, encouragement to participate, time savings, reduced embarrassment, pain perception, facilitation of medical follow-up, and willingness to recommend screening to others. Response options were "Yes," "Maybe," and "No." The questionnaire was administered after sample collection.

### Statistical analysis

Participant sociodemographic characteristics, cytological findings, and HPV DNA results were recorded. HPV positivity rates, sample adequacy, and participant satisfaction were compared between the clinician-collected and self-collected groups. Statistical analyses were performed by International Business Machines Statistical Package for the Social Sciences (IBM SPSS) Statistics for Windows, Version 26.0 (IBM Corp., Armonk, NY, USA). Chi-square tests analyzed categorical variables and Student's t-test analyzed continuous variables. A formal a priori power analysis was not performed. A post-hoc power analysis based on the observed HPV positivity rates (27.5 vs. 17.8%; two-sided chi-square test, α=0.05) indicated a statistical power of approximately 24%. Statistical significance was set at p<0.05.

## RESULTS

### Demographic and clinical characteristics

No statistically significant differences were observed in most demographic and clinical characteristics between the control (n=51) and self-collection (n=73) groups (Table 1). The mean age was 41.08±7.57 years in the control group and 42.73±7.15 years in the self-collection group (p=0.220). Median parity was 2 (range 0–4) in both groups (p=0.766). The majority of participants were married (86.3 vs. 84.9%) and sexually active (84.3 vs. 84.9%).

**Table 1:** Demographic and clinical characteristics of control and self-collection groups.

Variable		Control (n=51)	Self-collection (n=73)	p-value
Age (years), mean±SD		41.08±7.57	42.73±7.15	0.220†
Number of children, median (range)		2 (0–4)	2 (0–4)	0.766‡
Reason for visit, n (%)				
	Bleeding	11 (21.6)	4 (5.5)	<0.001§
	Discharge	8 (15.7)	7 (9.6)	
	Desire for pregnancy	5 (9.8)	1 (1.4)	
	Abdominal pain	9 (17.6)	3 (4.1)	
	Routine examination¶	16 (31.4)	55 (75.3)	
	ncontinence	1 (2.0)	3 (4.1)	
	Hirsutism	1 (2.0)	0 (0.0)	
Marital status, n (%)				
	Married	44 (86.3)	62 (84.9)	0.835§
	Divorced	7 (13.7)	11 (15.1)	
Relationship status, n (%)				
	Not sexually active	8 (15.7)	11 (15.1)	0.925§
	Sexually active	43 (84.3)	62 (84.9)	
Previous screening, n (%)				
	No	21 (41.2)	42 (57.5)	0.073§
	Yes	30 (58.8)	31 (42.5)	
Employment status, n (%)				
	Not working	28 (54.9)	31 (42.5)	0.172§
	Working	23 (45.1)	42 (57.5)	
Vaginitis treatment history, n (%)				
	Not received	23 (45.1)	38 (52.1)	0.446§
	Received	28 (54.9)	35 (47.9)	
Contraceptive method, n (%)				
	None	8 (15.7)	7 (9.6)	0.227§
	Condom	15 (29.4)	30 (41.1)	
	Intrauterine device	10 (19.6)	11 (15.1)	
	Oral contraceptive	5 (9.8)	3 (4.1)	
	Tubal ligation	5 (9.8)	3 (4.1)	
	Traditional methods	8 (15.7)	19 (26.0)	
Income level, n (%)				
	Below minimum wage	21 (41.2)	18 (24.7)	0.146§
	Minimum wage	16 (31.4)	28 (38.4)	
	Above minimum wage	14 (27.5)	27 (37.0)	
Smoking status, n (%)				
	Non-smoker	39 (76.5)	49 (67.1)	0.259§
	Smoker	12 (23.5)	24 (32.9)	

Note: Control group: clinician-collected cervical samples; Self-collection group: self-collected cervicovaginal samples. SD: standard deviation.

† Student's t-test.

‡ Mann-Whitney U test.

§ Chi-square test.

¶ Post-hoc comparison for routine examination: p=0.005.

The reason for clinic visit differed significantly between groups (p<0.001). The majority of participants in the self-collection group (75.3%) presented for routine screening, whereas only 31.4% of the control group presented for this reason. In the control group, the most common reasons for presentation were symptomatic complaints: bleeding (21.6%), abdominal pain (17.6%), and discharge (15.7%) (post-hoc comparison for routine examination: p=0.005).

Regarding screening history, 57.5% of participants in the self-collection group had never been screened, compared with 41.2% in the control group, representing a borderline significant difference (p=0.073). No significant differences were detected between groups for marital status, relationship status, employment status, income level, contraceptive method use, vaginitis treatment history, or smoking status (all p>0.05). Although 41.2% of control group participants earned below minimum wage compared with 24.7% in the self-collection group, this difference was not statistically significant (p=0.146).

### Human papillomavirus and cytological findings

Overall HPV positivity was 21.8% (27/124). Group-specific rates were 27.5% (14/51) in the control group and 17.8% (13/73) in the self-collection group, a difference that was not statistically significant (p=0.200).

Cytology results were negative for malignancy in 100% of the control group and 94.5% of the self-collection group (p=0.236). Abnormal cytology results were detected exclusively in the self-collection group (5.5%), comprising three cases of atypical squamous cells of undetermined significance (ASC-US) (4.1%) and one case of atypical squamous cells, cannot exclude high-grade squamous intraepithelial lesion (ASC-H) (1.4%).

Inflammatory cytological changes were the most common smear finding, observed in 60.8% of control group samples and 57.5% of self-collection group samples (p=0.768). The distribution of other findings was similar between groups: non-inflammatory smears (31.4 vs. 37.0%), bacterial vaginosis (5.9 vs. 2.7%), and atrophy (2.0 vs. 2.7%) (Table 2).

**Table 2:** Human papillomavirus results, cytological findings, and microbiological profiles in control and self-collection groups.

Variable		Control (n=51)	Self-collection (n=73)	p-value†
HPV status, n (%)				
	Negative	37 (72.5)	60 (82.2)	0.200
	Positive	14 (27.5)	13 (17.8)	
Cytology result, n (%)				
	Negative for malignancy	51 (100.0)	69 (94.5)	0.236
	ASC-US	0 (0.0)	3 (4.1)	
	ASC-H	0 (0.0)	1 (1.4)	
Smear findings, n (%)				
	No inflammation	16 (31.4)	27 (37.0)	0.768
	Inflammation present	31 (60.8)	42 (57.5)	
	Bacterial vaginosis	3 (5.9)	2 (2.7)	
	Atrophy	1 (2.0)	2 (2.7)	
*Candida status*, n (%)				
	Negative	46 (90.2)	67 (91.8)	0.760
	Positive	5 (9.8)	6 (8.2)	
*Trichomonas vaginalis* status, n (%)				
	Negative	51 (100.0)	72 (98.6)	0.401
	Positive	0 (0.0)	1 (1.4)	

Note: ASC-H: atypical squamous cells, cannot exclude high-grade squamous intraepithelial lesion; ASC-US: atypical squamous cells of undetermined significance; HPV: human papillomavirus. Control group: clinician-collected cervical samples obtained under direct visualization during speculum examination; Self-collection group: self-collected cervicovaginal samples obtained by participants following standardized instruction.

† All comparisons performed by the chi-square test (Fisher's exact test used for small cell counts).

### Infectious agent detection

*Candida* species were detected in five participants (9.8%) in the control group and six participants (8.2%) in the self-collection group (p=0.760). *Trichomonas vaginalis* was identified in a single participant (1.4%) in the self-collection group (p=0.401).

### Participant satisfaction

The mean satisfaction score in the self-collection group was 12.6±1.62 out of a maximum of 16 (median=12; range=9–16), corresponding to 78.8% positive responses. The minimum observed score was 9.

## DISCUSSION

This prospective controlled clinical trial provides evidence that self-collected cervicovaginal HPV samples are diagnostically comparable to clinician-collected cervical samples. We observed no significant differences in HPV positivity rates (17.8 vs. 27.5%), cytological abnormalities, or infectious findings between groups, while participant satisfaction scores averaged 78.8% positive responses. These findings support self-collection as a reliable and feasible alternative for cervical cancer screening.

Recent systematic reviews and meta-analyses have demonstrated that HPV self-sampling strategies significantly increase participation in cervical cancer screening programs. A 2024 systematic review and meta-analysis by Wong et al., comprising 94,908 women predominantly from Europe and North America, reported that self-sampling strategies markedly improved participation rates compared with clinician-based screening, particularly among hard-to-reach populations and non-attendees^10^. Our finding that 57.5% of self-collection participants had never been screened, as compared with 41.2% in the control group (p=0.073), aligns with this evidence, suggesting that self-sampling may preferentially attract previously unscreened women. The majority of participants in the self-collection group reported that the method enhanced privacy, increased motivation for medical consultation, and encouraged them to recommend screening to others. These findings suggest that self-sampling may reduce psychosocial barriers, such as embarrassment and shame, thereby expanding the reach of cervical cancer screening programs.

Studies evaluating the diagnostic accuracy of sampling methods for HPV screening indicate that self-sampling can achieve clinically comparable performance to clinician-collected samples. According to recent meta-analyses, self-sampling performs well for HPV detection in general, but may be less sensitive than clinician-collected sampling for identifying high-grade cervical lesions. This limitation should be taken into account when assessing the clinical consequences of self-sampling-based screening techniques^11^. A meta-analysis of 16 studies by Phillips et al. reported higher sensitivity for self-collected HPV samples compared with clinician-collected cytology, emphasizing that HPV-based screening permits longer and safer screening intervals^12^. While these findings establish the superiority of HPV-based screening over cytology, our observation of no significant difference in HPV positivity between self-collected and clinician-collected HPV samples supports self-sampling as a reliable method within HPV-based screening strategies. The 9.7 percentage-point difference in HPV positivity observed in this study (27.5 vs. 17.8%) likely reflects differences in clinical presentation between groups rather than sampling method performance, as the control group comprised predominantly symptomatic women. Self-collection thus represents not only a diagnostically accurate approach but also one that may enhance the accessibility of HPV-based screening in clinical practice.

An additional methodological consideration is the baseline difference in clinical presentation between the two groups. Women in the clinician-collected group more frequently presented with symptoms such as bleeding, pain, or discharge, whereas the self-collection group consisted predominantly of women attending routine screening. This imbalance reflects real-world clinical practice but may also influence HPV positivity rates. Therefore, differences observed between groups should be interpreted cautiously, as they may partly reflect underlying patient characteristics rather than the sampling method itself.

A nonrandomized clinical trial of 599 participants comparing self-collected and clinician-collected samples demonstrated high concordance for high-risk HPV detection and similar diagnostic performance for detecting clinically significant cervical lesions, such as CIN2+^13^. Although the present study did not include histopathological outcomes, the detection of four cytological abnormalities (three ASC-US and one ASC-H) exclusively in the self-collection group, despite lower overall HPV positivity, demonstrates that cervicovaginal self-sampling can identify clinically relevant cellular changes. These findings indicate that self-collected HPV sampling is reliable for high-risk HPV detection and can be effectively integrated into cervical cancer screening with appropriate triage approaches. These findings should be interpreted cautiously, but they suggest that self-collected sampling may have potential within HPV-based screening strategies. In this setting, biomarker-based triage could improve the clinical usefulness of self-sampling. Lorenzi et al. found that combining a positive HPV test with positive p16/Ki-67 dual staining in self-sampled vaginal material accurately identified women at higher risk of cervical cancer^14^.

A review spanning the years 2000–2024 reported that self-collected samples demonstrate comparable accuracy to clinician-collected samples for diagnosing *Candida, Trichomonas vaginalis*, and other vaginal infections, presenting a feasible method particularly in resource-limited settings^15^. Our detection rates for *Candida* (8.2–9.8%) and *Trichomonas vaginalis* (0–1.4%) were comparable between self-collected and clinician-collected samples, with no significant differences. Although the primary objective was HPV DNA detection, these findings suggest that self-sampling may be applicable not only for HPV screening but also for concurrent infectious evaluation. However, the role of self-collection in infection diagnosis was a secondary finding in this study and should be interpreted within the context of HPV screening.

### Limitations

First limitation was the relatively small sample size (n=124) as it may have reduced the likelihood of detecting rare cytological abnormalities and high-grade lesions. Second, the absence of histopathological verification and long-term clinical follow-up data limits the assessment of self-sampling performance for predicting high-grade cervical lesions. Third, a significant baseline difference in the reason for clinic presentation constitutes a major confounder. The self-collection group comprised predominantly asymptomatic women seeking routine screening, whereas the control group consisted largely of symptomatic women presenting with bleeding or pain. This imbalance may have contributed to the higher HPV positivity observed in the control group and limits the direct comparison of sampling method performance. Fourth, since participants in the self-collection group were actively involved in the educational process regarding the sampling method, the high acceptability and satisfaction levels observed may differ from those in real-world screening programs where such intense support may not be available. Fifth, we acknowledge that the lack of prior sample size calculation constitutes a methodological limitation. The post-hoc power analysis revealed approximately 24% statistical power, indicating that the study did not have sufficient power to detect the observed difference in HPV positivity rates. Future studies should include formal pre-power analyses with larger sample sizes, and the satisfaction questionnaire was developed by the investigators without prior psychometric validation. The absence of internal consistency analysis limits the reliability assessment of this instrument. Finally, as this was a single-center study, multicenter trials with larger sample sizes are required to generalize these findings to populations with diverse sociocultural backgrounds and healthcare infrastructures.

## CONCLUSION

Self-collected HPV samples yield diagnostically comparable results to clinician-collected specimens. Self-collected HPV samples demonstrated similar HPV detection rates compared with clinician-collected specimens in this study. Given its ease of use and enhanced comfort, self-sampling represents a feasible alternative sampling method to facilitate participation, particularly in regions with limited healthcare access. Integrating this approach into Turkey's national infrastructure could enhance community-based screening effectiveness and early detection. Future research requires larger-scale, multicenter trials and the development of robust follow-up systems for screen-positive patients.

## Data Availability

The datasets generated and/or analyzed during the current study are available from the corresponding author upon reasonable request.
